# Polyploidization and genomic selection integration for grapevine breeding: a perspective

**DOI:** 10.3389/fpls.2023.1248978

**Published:** 2023-11-15

**Authors:** Rohit Bharati, Madhab Kumar Sen, Lucie Severová, Roman Svoboda, Eloy Fernández-Cusimamani

**Affiliations:** ^1^ Department of Crop Sciences and Agroforestry, The Faculty of Tropical AgriSciences, Czech University of Life Sciences Prague, Suchdol, Czechia; ^2^ Department of Agroecology and Crop Production, Faculty of Agrobiology, Food and Natural Resources, Czech University of Life Sciences Prague, Suchdol, Czechia; ^3^ Department of Economic Theories, Faculty of Economics and Management, Czech University of Life Sciences Prague, Prague, Czechia

**Keywords:** genomic selection, grapevine, *in vitro*, omics, plant breeding, polyploidization

## Abstract

Grapevines are economically important woody perennial crops widely cultivated for their fruits that are used for making wine, grape juice, raisins, and table grapes. However, grapevine production is constantly facing challenges due to climate change and the prevalence of pests and diseases, causing yield reduction, lower fruit quality, and financial losses. To ease the burden, continuous crop improvement to develop superior grape genotypes with desirable traits is imperative. Polyploidization has emerged as a promising tool to generate genotypes with novel genetic combinations that can confer desirable traits such as enhanced organ size, improved fruit quality, and increased resistance to both biotic and abiotic stresses. While previous studies have shown high polyploid induction rates in *Vitis* spp., rigorous screening of genotypes among the produced polyploids to identify those exhibiting desired traits remains a major bottleneck. In this perspective, we propose the integration of the genomic selection approach with omics data to predict genotypes with desirable traits among the vast unique individuals generated through polyploidization. This integrated approach can be a powerful tool for accelerating the breeding of grapevines to develop novel and improved grapevine varieties.

## 1 Introduction

Grapevines (*Vitis* spp.) are woody perennial crops belonging to the Vitaceae family. These are extensively cultivated for their fruits, which are used in wine production, along with for grape juice, raisins, and table grapes. The wine industry has a substantial influence on the global economy. Additionally, grapes also contain beneficial compounds, such as resveratrol and flavonoids, which have been shown to have antioxidant, anti-inflammatory, and anti-cancer properties and help prevent chronic diseases (Sabra et al., 2021; Zhou et al., 2022; Cuciniello et al., 2023). Currently, grapevine breeding is facing several environmental challenges such as unforeseen climate change and pervasiveness of diseases and pests (Marín et al., 2021; Töpfer and Trapp, 2022). On the other hand, pests and diseases lead to substantial yield losses and abridged fruit quality. To overcome these challenges, grapevine breeders develop drought-tolerant or disease-resistant varieties. However, producing these varieties via traditional breeding methods can be an extensive, lengthy, and complex process. One possible alternative to these traditional breeding methods can be artificial polyploidization. In the context of grapevine breeding, artificially increasing the number of sets of chromosomes and creating a polyploid can be a promising tool to generate genotypes with novel genetic combinations not present in the parental lines. Polyploidization can confer agronomically desirable traits, such as enhanced organ size, improved fruit quality, and increased resistance to both biotic and abiotic stresses (Touchell et al., 2020; Gantait and Mukherjee, 2021; Beranová et al., 2022; Jin et al., 2022; Bharati et al., 2023). Furthermore, this method offers a range of advantages over traditional breeding techniques, such as rapid production of polyploid individuals, increased genetic diversity, cost-effectiveness, and applicability across a broad spectrum of plant species.

While synthetic polyploidization has proven to be a potent tool in breeding various plants, its full potential in grapevine breeding remains untapped. However, there are some plants such as *Anemone sylvestris* (Šedivá et al., 2019), *Thymus vulgaris* (Homaidan Shmeit et al., 2020), and *Lycium ruthenicum* (Rao et al., 2020), where polyploidation has been used previously. Synthetic polyploidization can quickly generate a high frequency of polyploids, however, it necessitates meticulous genotype screening to screen for desired traits. Genotype screening after polyploidization may be more straightforward for crops with shorter life cycles or those that exhibit early expression of desired traits, such as herbs. However, when it comes to perennial crops like grapevines, this process demands substantial labor and financial investments. This could potentially explain the limited research on screening genotypes with desired agronomic traits, such as increased yield and tolerance to abiotic and biotic stress, after polyploidization in any *Vitis* species.

The genomic selection (GS) of the produced polyploids can be an interesting option, while predicting the desirable genotypes following the artificial polyploidization. In general, GS involves using genomic information to predict the breeding value of plants and selecting the best individuals with desired traits of interest for further breeding (Newell and Jannink, 2014; Bhat et al., 2016; Crossa et al., 2017). In the context of grapevine breeding, the breeders can easily envisage the genetic potential of an individual polyploid plant for a given trait, bypassing the time-consuming and labor-intensive screening methods. In the current perspective, we will discuss the current state of the polyploidization in the grapevines towards crop improvement. Additionally, we aim to identify the potential of polyploidization and GS integration towards predictive breeding of grapevines. This integrated approach can be a powerful tool for accelerating the breeding of grapevines to develop novel and improved grapevine varieties. This will not only help breeders obtain genotypes with high agronomic value but will also reduce the time, labor, and capital investments that would otherwise become futile if poorly performing genotypes are obtained.

## 2 Polyploidization in grapevine improvement: current status and limitations

Polyploidization has been used as a tool for crop improvement for many years. To date, this technique has been successfully used in many species to obtain traits such as increased fruit size, enhanced disease resistance, and tolerance to a variety of stresses (Šedivá et al., 2019; Homaidan Shmeit et al., 2020; Rao et al., 2020). Recent studies have focused on inducing polyploids in *Vitis* through various methods and anti-mitotic agents. *In vivo* methods, which involve treating the entire plant or a part of a plant, have been attempted for polyploidization in *Vitis* species, but have remained less effective compared to *in vitro* methods. For example, in a study by Kara et al. (2018), the use of colchicine treatment in grape genotypes resulted in no tetraploid plants being identified, except for one grape cv (Trakya İlkeren), which showed aneuploidy at a specific concentration of colchicine. Similarly, in another study by (Kara and Doğan, 2022), the application of oryzalin and N_2_O to cuttings of 41B Chasselas and Fercal (*Vitis vinifera* L.) rootstocks did not result in the production of any polyploid individuals through *in vivo* methods. Kara and Yazar (2021) also examined changes in stomata guard cells but found no differences at the ploidy level. More recently, Kara and Doğan (2023) utilized Oryzalin and N_2_O to treat a total of 1200 plants belonging to two grapevine cultivars, yielding only one tetraploid genotype for each cultivar. These results suggest that *in vivo* methods for polyploidization in *Vitis* species are not an effective approach in obtaining high frequency polyploids.

Recent progress with *in vitro* methods offers a promising avenue for inducing polyploids in many *Vitis* species. Acanda et al. (2015) found that the *in vitro* treatment of colchicine at a concentration of 0.2% was most effective for producing tetraploid plantlets in *Vitis*, with a tetraploid rate of 25%. Xie et al. (2015) also achieved successful polyploidization in *Vitis* through colchicine treatment, with a polyploid induction rate of 37.78%, when pre-embryogenic calli were used. Additionally, Sinski et al. (2014) found that both colchicine and oryzalin were effective in inducing polyploids in *Vitis*. Although, oryzalin was found to be more effective than colchicine in polyploid induction efficiency in *Vitis* spp., ranging between 1.66-10.5% compared to 3.2-5% (**Table 1**). These recent developments in polyploidization techniques in *Vitis* species could have significant implications for improving crop yield and quality in viticulture. Interestingly, polyploidization in *Vitis* species under *in vitro* conditions without the use of anti-mitotic agents has also been observed. Catalano et al. (2021) regenerated grapevine plants via somatic embryogenesis and observed a 9% tetraploid induction rate, even though no anti-mitotic agents were used to induce polyploidization. The overall findings of the studies suggest that *in vitro* chromosome doubling could be a viable approach to generate polyploid grapevines with desirable characteristics, which could potentially have significant implications for the grape industry. A list of major attempts to induce polyploids in a number of *Vitis* species has been summarized in **Table 1**.

**Table 1:** List of major artificial polyploidization attempts in various *Vitis* Spp.

Reference	*Vitis* species	Anti-mitotic agent used	Mode of treatment	Findings
Kara and Doğan (2023)	41 B [Chasselas (*Vitis vinifera* L.) × (Vitis berlandieri Planch.)] and Fercal [(*Vitis vinifera* L. × *Vitis berlandieri*) × 333 EM (Cabernet-Sauvignon ×*Vitis berlandieri*)]	Oryzalin and N_2_O	*in vivo*	A total of 1200 plants for each genotype and each anti-mitotic agent were used for polyploid induction. For the 41 B genotype, one mixoploid plant and one tetraploid plant were obtained. For the Fercal genotype, four mixoploid plants and one tetraploid plant were obtained.
Kara and Doğan (2022)	41 B Chasselas (*Vitis vinifera* L. × *Vitis berlandieri* Planch) and Fercal [(*Vitis vinifera* x *Vitis berlandieri*) × 333 EM]	Oryzalin and N_2_O	*in vivo*	The application of oryzalin and N2O to cuttings of 41B and Fercal rootstocks did not result in the production of any polyploid individuals through *in vivo* methods.
Kara and Yazar, 2022	*Vitis vinifera* L. (Ekşi Kara & Trakya İlkeren)	Colchicine (were applied to meristematic part of seedling twice a day (in 8.30 and 18:00) for 3 days)	*in vivo*	The viability of shoot tips varied among the cultivars and decreased with increasing colchicine doses, except for the application of 1 g L-1 on ‘Ekşi Kara’ and 5 g L-1 on ‘Trakya İlkeren’. In ‘Ekşi Kara’, the lowest shoot tip viability rates compared to the control (100%) were observed at doses of 4 g L-1 (31.77%), 6 g L-1 (47.26%), and 3 g L-1 (51.84%). Colchicine was administered to seedlings from two grape cultivars, resulting in polyploidy induction, depending on the application methods and genotypes. 5 mg/L was found to be effective for Ekşi Kara and 6 mg/L was effective for Trakya İlkeren seedlings.
Kara and Yazar, 2021	*Vitis vinifera* L.	Colchicine	*in vivo*	Examination through chloroplast counts and FC analyses of stoma guard cells revealed that these changes did not result in any differences at the genomic level.
Catalano et al., 2021	*Vitis vinifera* L. (Catarratto, Frappato, and Nero d’Avola)	none	*in vitro*	Grapevine plants regenerated via somatic embryogenesis in this study observed a nine percent tetraploid induction rate.
Kara and Yazar, 2020	Ekşi Kara (*Vitis vinifera* L.)	Colchicine	*in vivo*	Eight different colchicine concentrations (0, 1, 2, 3, 4, 5, and 7.5 g L-1) were administered twice daily (at 8:30 AM and 6:00 PM) to the meristematic part of seedlings for a duration of 3 days, starting when the first true leaves appeared. Although, flow cytometric analysis confirmed that no polyploids were obtained indicating the employed approach was ineffective.
Kara et al., 2020	41 B Chasselas (*Vitis vinifera* L. × *Vitis berlandieri* Planch) and ‘Trakya İlkeren’, ‘ Gök Üzüm’ and ‘Ekşi Kara’ grape cultivars (*Vitis vinifera* L.)	N_2_O	*in vivo*	Flowcytometric analysis confirmed that the application of N_2_O failed in polyploidy induction in grapevine genotypes used.
Kara et al., 2018	*41 B Chasselas (Vitis vinifera L.) x* *(Vitis berlandieri Planch.), Gök Üzüm (Vitis vinifera* *L.), Trakya İlkeren (Vitis vinifera L.)*	Colchicine	*in vivo*	The use of colchicine treatment in grape genotypes used by authors revealed that all untreated seedlings had diploid ploidy levels (2n=2x=38), and no tetraploid plants were identified. Only the grape cv Trakya İlkeren responded to the colchicine treatment, inducing aneuploidy at a concentration of 5 gL-1, resulting in a ploidy level of 2n=2x=40.
Xie et al., 2015	*Vitis X Muscadinia*	Colchicine and oryzalin	*in vitro*	This research accomplished the successful generation of a significant proportion of tetraploid plants from hybrids of 101-14 Mgt *X M. rotundifolia* cv. Trayshed. Colchicine treatment was found most effective, with the highest polyploid induction rate of 37.78% when pre-embryogenic calli were used for treatment.
Acanda et al., 2015	*Vitis vinifera* L. cv. Mencı´a	Colchicine	*in vitro*	In this study, the most effective concentration of colchicine for producing tetraploid plantlets was found to be 0.2%, resulting in a tetraploid rate of 25%. No mixoploid or chimeric plantlets were observed during the experiment.
Chang et al., 2014	Victoria grape (*Vitis vinifera* L.)	Colchicine	*in vitro*	The most effective method to enhance chromosome duplication efficiency was observed by treating the third and fourth buds with 0.05% colchicine for 48 hours or 0.1% colchicine for 24 hours. The primary generation cells displayed doubling rates of 33% and 31% respectively.
Sinski et al., 2014	*Vitis vinifera* L. (Crimson seedless and BRS Clara)	Colchicine and oryzalin	*in vitro*	In this study, colchicine and oryzalin were both effective in inducing polyploids. Oryzalin was found to be more effective in polyploid induction efficiency ranging between 1.66-10.5%, than colchicine with 3.2-5%.
Yang et al., 2006	*Vitis vinifera* L. cv. Sinsaut	Colchicine	*in vitro*	A total of 29 plantlets generated from embryos treated with colchicine were examined. Out of the 29 plantlets, five (which constituted 17.2%) were found to be tetraploid (2n = 2x = 76), while all the remaining plantlets were diploid (2n = 2x = 38). The application of colchicine to somatic embryos did not result in the production of any chimeras.
Notsuka et al., 2000	*Vitis vinifera* L. and American hybrids	Colchicine	*in vitro*	Axillary buds of growing shoots were used to perform *in vitro* chromosome doubling on 29 diploid, 3 triploid, and 1 tetraploid grape accession of Vitis spp. The success rates varied among the accessions, with the range being from 6% (for ‘Hakata White’) to 47% (for ‘Pusa Seedless’) in V. vinifera, and from 4% (for ‘Fuefuki’) to 35% (for ‘Prima Seedless’) in the American species.

While the successful induction of polyploids in grapevines has been documented in several studies, only a few have assessed the resulting population for desirable agronomical traits, where these assessments have primarily focused on stomatal and leaf characteristics (Yang et al., 2006; Sinski et al., 2014; Xie et al., 2015; Kara et al., 2018; Kara et al., 2020; Catalano et al., 2021; Kara and Yazar, 2021; Kara and Doğan, 2022; Kara and Yazar, 2022). A study has also delved into epigenetic regulation through DNA methylation, shedding light on how changes in DNA methylation patterns can impact gene expression and phenotypic traits in polyploid grapevine (Xiang et al., 2023). Surprisingly, the essential agronomical traits with economic value, including vigor, yield, berry size, berry color, Brix levels, as well as ripening period, have received minimal attention in this context. One notable exception is the study by Notsuka et al. (2000), which comprehensively evaluated the generated polyploids with a specific emphasis on grape-related traits, recognizing that fruit-related traits require a more substantial investment of time and effort. In this study, the authors explored the potential of *in vitro* chromosome doubling across 29 diploid, 3 triploid, and 1 tetraploid grape accession of *Vitis* spp., successfully achieving high polyploid inductions of up to 47%. Subsequent field trials of these polyploids unveiled a diverse range of desirable traits, including vigorous growth, improvements in skin color, and enhanced berry size. However, it is noteworthy that the performance of induced polyploids varied significantly depending on the cultivar. In some cases, the induced polyploids exhibited no significant changes and were akin to the source genotypes. These findings suggest that the strategy of individually subjecting each polyploid to phenotypic screening for desired traits may not be an efficient and economical approach. The uncertainty in phenotypic outcomes highlights the immediate need to enhance our ability to control and refine genotype screening processes post-polyploidization.

With the advent of sequencing technologies, such as genomics, and the discovery of markers associated with genes/QTLs of interest, a more indirect selection and screening method called Marker-assisted selection (MAS) has emerged (Xu and Crouch, 2008; Ben-Ari and Lavi, 2012). MAS offers a promising solution for screening genotypes with desired traits after polyploidization, where specific molecular markers can be utilized to identify genotypes possessing the desired traits. Nonetheless, the presence of complexities, such as genome duplication, can present challenges when developing markers closely linked to the desired traits (Crossa et al., 2017). Moreover, MAS usually relies on a handful of loci with significant effects, which might fail to encompass the complete range of genetic variations accountable for the trait in question (Ben-Ari and Lavi, 2012; Olatoye et al., 2019). The influence of genome duplication further complicates the situation. Therefore, accurately predicting the performance of a polyploid genotype based solely on its molecular markers can be a challenging task.

As compared to MAS, a more promising approach for polyploid screening generated from polyploidization could be GS, which uses genomic information to predict the performance of plants, enabling breeders to select desirable traits more efficiently and accurately (Newell and Jannink, 2014; Bhat et al., 2016). GS has been shown to be more effective than MAS in identifying desirable genotypes due to its enhanced accuracy, reduced reliance on specific markers, incorporation of non-additive effects, and reduced cost and time (Lorenz et al., 2011; Jonas and De Koning, 2013; Crossa et al., 2017). However, the predictive accuracy of the employed model is crucial for the effectiveness of GS, thus, careful selection and optimization of the prediction model are necessary to ensure its effectiveness.

## 3 Choosing the best individual: omics based genomic selection for polyploid screening

In general, GS includes all the genomics-driven strategies to select the best individuals from a testing population (TE) for breeding. The TE and the training populations (TR) are the key components of any genomic selection process. While TE refers to individuals with only genomic data, TR includes the group of individuals for whom both genomic and phenotypic data are available. In the current context, the individuals generated via polyploidization following genotyping will serve as the TE. Previously, traditional techniques such as PCR-based techniques and single nucleotide polymorphism (SNP) genotyping arrays have been used (Viana et al., 2016; Brault et al., 2021; Brault et al., 2022). However, in recent years, with the significant advancement and reduction in sequencing costs, crop breeders have shifted their focus towards omics-based strategies. The main advantage of multi-omics data in the genomic selection approach is its ability to enhance prediction accuracy by capturing diverse molecular interactions and factors influencing phenotypic traits (Ye et al., 2020; Sen et al., 2023). Although utilization of multi-omics data for GS in grapevine is lacking, transcriptome and metabolome data have been used for GS in maize breeding (Guo et al., 2016; Westhues et al., 2017).

Omics data such as genomics, transcriptomics, and metabolomics can provide valuable as well as novel insights on how to improve the precision of genomic relationship estimation in polyploids. Since the availability of the grapevine genome (initially in 2007 and later re-sequenced in 2019), omics-based studies have been extensively used to study polyploidy and heterozygosity in grapevines (The French–Italian Public Consortium for Grapevine Genome Characterization, 2007; Liang et al., 2019). For example, in a recent study conducted by (Han et al., 2023), the authors used reference genome-based RNA-seq data analysis to identify the probable pathways involved in the freezing response in grapevines. Likewise, a few studies were conducted on grapevine breeding using various omics technologies (Wang et al., 2021; Savoi et al., 2022). In addition to genomics, metabolomics, and transcriptomics, derived and innovative omics (such as epigenomics and epitranscriptomics) can also be used for comprehensive understanding of the complex epigenetic modifications in the induced polyploids. Recently, there has been growing recognition of the important roles of epigenetic regulations and memories in the stress response of crops, including grapevines (Atanassov et al., 2022; Dal Santo et al., 2022; Jia et al., 2023). Epitranscriptomics, which deals with chemical modifications on RNA molecules, is yet to be applied in viticulture. However, there are some instances where epitranscriptomic study has been used for crop improvement (Hou and Wan, 2021). Nevertheless, despite the fact that these omics technologies can provide substantial insights into the molecular functioning of the genes of interest in grapes, they have several disadvantages, such as an inadequate view of biological processes. Crop traits and performance depend on multifaceted interactions between different biological components. Hence, the results obtained from single omics may miss the full system-level understanding required for effective understanding of the novel and influenced traits among the polyploid population. In this scenario, we recommend using more informative multi-omics data to get a comprehensive understanding of the grapevine traits and their genetic basis prior to TE selection. While multi-omics for breeding purposes grapevine are limited, these strategies are extensively used in other plant species such as rice (Sun et al., 2022), and maize (Farooqi et al., 2022). More details concerning the multi-omics in plant breeding can be found in Mahmood et al., 2022. In the context of grapevine breeding, data derived from multi-omics analyses can be used to identify the major genes that might enhance environmental adaptation and aid in the selection of crucial agronomic traits. Inferring the exact link between the genes and the final phenotype might be difficult due to the lack of middle omics (from genomics to phenomics). Integration of genome-wide association studies (GWAS) with other omics (such as metabolomics and transcriptomics) will reduce the variety of candidate genes and aid system analysis of gene function. For instance, GWAS integration with transcriptome-wide association studies (TWAS) can be used to discover expression QTLs (eQTLs) (fine-mapping technique) in the induced polyploid grapevines. This approach can be an excellent option to establish the relationship between transcript abundance and phenotypic variance while simultaneously gaining insights into the regulatory functions of genetic variations responsible for phenotypic changes. Earlier, the GWAS-TWAS integrative approach was used in rice (Anacleto et al., 2019; Mahmood et al., 2022) and cotton (Li et al., 2020; Mahmood et al., 2022). Combined GWAS and metabolome-wide association studies (MWAS) can simultaneously screen a vast number of grapevine accessions for possible associations between their genomes and diverse metabolites. This collaborative approach will offer significant insights into the genetic basis of complex traits and the level of metabolic diversity within the population. Furthermore, the integration of the eQTLs and metabolite quantitative trait loci (mQTLs) can also complement GWAS while predicting the phenotypic outcomes of the induced polyploid genotypes. This integration contemplates the variations in mRNA expression and metabolite production and will provide novel insights into the eventual performance of the produced varieties in a comparatively short time period, which otherwise would be time-consuming. The multi-omics datasets can be integrated via correlation-based integration, network-based integration, and pathway-based integration. In the context of multi-omics data integration, correlation-based methods aim to identify patterns of co-expression across different omics datasets, whereas network-based integration focuses on creating biological networks representing various interactions between biomolecules, followed by the integration of omics data onto these networks. The pathway-based integration method focuses on mapping the omics data onto predefined biological pathways. Even though these methods have their own advantages and disadvantages, in practice, the choice of the appropriate method depends on the research question and the availability of data. Often, a combination of these methods can be used for a more comprehensive understanding at the organismal level. More details on systematic multi-omics data integration approaches can be found in Fabres et al., 2017 and Jamil et al., 2020.

**Figure 1:**
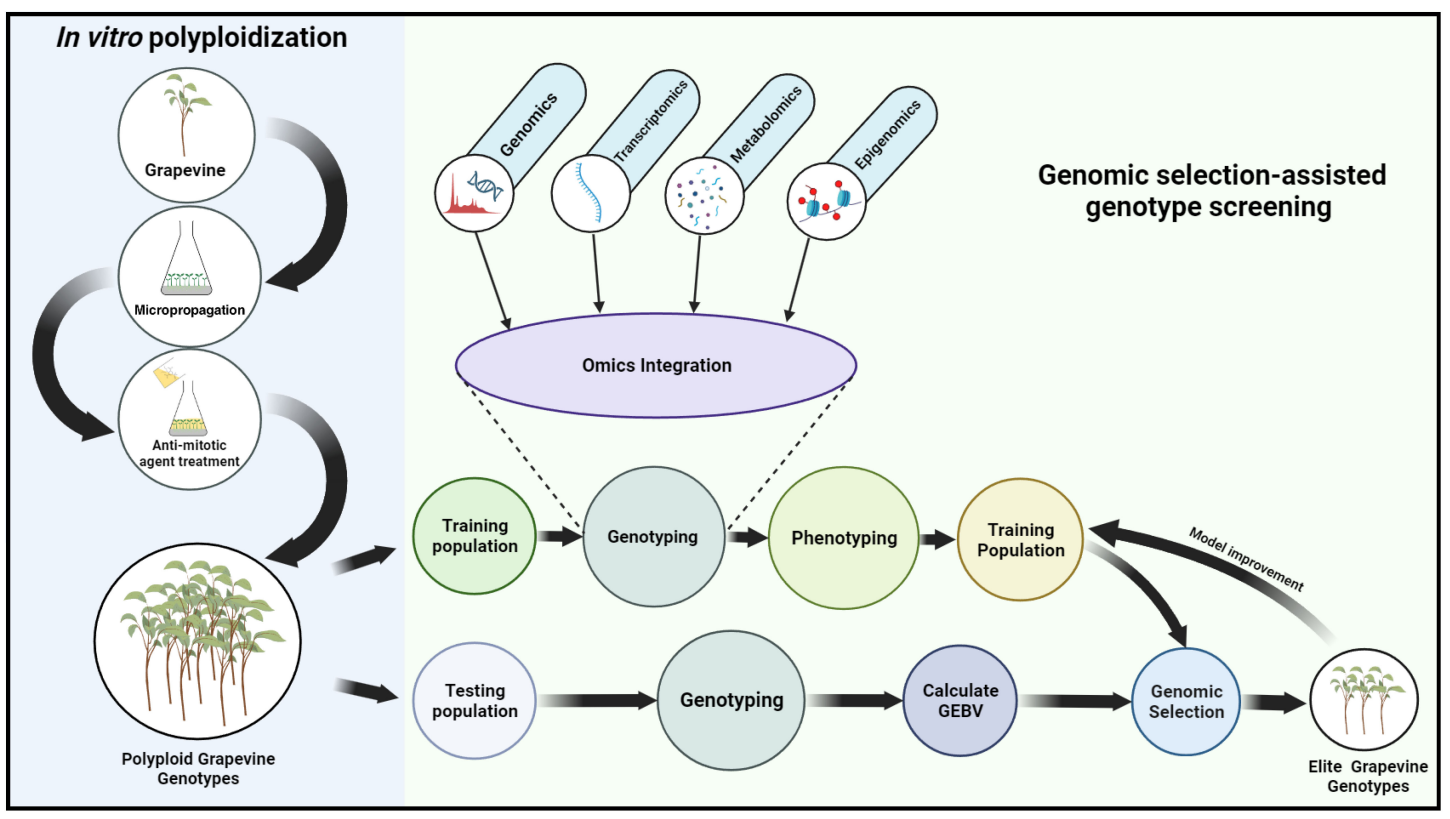
Flow diagram illustrating potential elite genotype screening via omics integrated genomic selection among polyploid population generated through *in vitro* polyploidization. The diagram depicts the various phases involved in genomic selection. By utilizing genotypic and phenotypic data acquired from the training population, the genomic selection models can be optimized, enabling the estimation of breeding values for superior genotypes.

The genotyping of the TR population is followed by its phenotyping. To ensure accurate phenotyping, it’s important to carefully design experiments and select appropriate traits to measure. Selection of traits and prioritization should be relevant to the goals of crop improvement, such as yield, disease resistance, drought tolerance, or nutritional quality. The previous GS studies on grapevines assessed various traits related to agronomical characteristics, drought tolerance, and yield components. These studies aimed to enhance understanding of these traits’ genetic architecture and identify molecular markers associated with their variation (Viana et al., 2016). Although data complexity increases, including more traits might provide a broader representation of phenotypic variation, allowing for a more comprehensive assessment of an individual’s genetic potential. For instance, (Flutre et al., 2022) phenotyped 279 *Vitis vinifera* training cultivars and assessed a total of 127 traits. Additionally, they also combined several other traits, making a total of 152. Despite using an extensive dataset, the study achieved high prediction accuracy for 50% of the response variables. Once phenotypic and genotypic data have been obtained from the TR population, they can be employed to construct prediction models, using phenotype as the response and genotype as the predictor. To date, several parametric models such as genomic best linear unbiased prediction (GBLUP), Bayesian regression-based methods (like BayesA), sparse linear mixed model methods (like BayesB), and Bayesian least absolute shrinkage and selection operator (BLASSO) methods (like BayesC) have been developed for GS. These models address different challenges and offer unique advantages. For a comprehensive understanding of these statistical models, one can refer to Budhlakoti et al. (2022). Previous GS studies in grapevines compared different prediction models and evaluated their performance. For example, Flutre et al. (2022) compared two multi-SNP models and determined that the dense RRBLUP/GBLUP model was a relevant default, while the sparse varbvs model achieved higher accuracy for traits closer to genetic variation. Brault et al. (2022) used Ridge Regression (RR) and LASSO models and found that predictive ability varied depending on the scenario and trait. In the current context, BLASSO could be an appropriate option as it provides a probabilistic framework that can accommodate uncertainty in variable selection, making it useful when dealing with multiple omics layers where interactions may be complex. Although it is important to note that each model has its strengths and weaknesses, the selection of a suitable model depends on specific objectives, genetic architecture, and available data. Comparisons and evaluations of different models are often recommended for optimal performance in GS. Following the model’s development, the next step is to select and validate the model. After a prediction model has been prepared and validated, it can be used to predict the Genomic Estimated Breeding Values (GEBVs) of individuals in the breeding population. The GEBVs can then be used as a parameter to rank individuals in the breeding population according to their predicted genetic merit for the trait of interest. **Figure 1** describes the potential screening of elite genotypes through omics-integrated genomic selection in a polyploid population generated via *in vitro* polyploidization.

## 4 Current challenges and the way forward

Artificial polyploidization in grapevines presents a bottleneck in plant breeding, but the use of GS for genotype screening following polyploidization offers a promising approach to address this issue. Although, for successful prediction of elite polyploid genotypes, it is crucial to consider the potential shortcomings to avoid or address them. Genotyping the produced grape polyploids via omics-based GS can introduce unique challenges, such as integration of the multi-omics data along with their proper management and interpretation and functional annotation and biological relevance of omics markers. Multi-omics datasets, which are often produced using diverse technologies and platforms, lead to data heterogeneity, besides producing a high-dimensional and complex data landscape. Hence, despite being informative and precise, the volume and complexity of the data make its management difficult, which might require dedicated tools and algorithms which are competent of handling the dimensionality and complexity of multi-omics data. In addition to these, multi-omics datasets usually contain a large number of variables and features, which complicates the downstream analyses. Besides data complexity, we also recommend focusing on reference genome availability and trait-marker associations. Alongside reference genome availability, detection of the biologically significant links between omics markers and complex traits in polyploids can also be intricate.

Synthetic polyploids are known to have better adaptability to a wide range of environments compared to their diploid counterparts. However, it is important to assess the Genotype × Environment (G×E) interactions, as they can greatly influence the predictive potential of GS (Mulder, 2016; Jarquín et al., 2017). Additionally, G×E is particularly relevant in crops such as grapevines, which are highly sensitive to environmental factors that could influence both the quantitative and qualitative characteristics of the crop (Dinu et al., 2021; Dal Santo et al., 2022). Another potential challenge is that, while traditional models show success in prediction, they often overlook vital non-additive effects like genomic imprinting and epistasis, impacting prediction accuracy (Jackson and Chen, 2010; Endelman et al., 2018; Varona et al., 2018; Hunt et al., 2020). Artificially induced polyploids exhibit substantial non-additive effects on phenotype, particularly notable in grapevines propagated by cutting and grafting, influencing traits and stress responses (Tan et al., 2023). Understanding and utilizing these additive effects is crucial for effective genomic prediction in grapevine breeding. For that, machine learning models like random forests, support vector machines, and deep neural networks could be instrumental due to their ability to capture complex marker-trait relationships, select markers, and handle noise (Heslot et al., 2014; Crossa et al., 2017; Wang et al., 2018; Van Dijk et al., 2021). Despite several challenges, integrated polyploidization and GS strategy could be an excellent option for grapevine breeding. An updated and detailed understanding of the associated challenges will be the main key. Active collaboration between the experts in genomics, bioinformatics, statistical genetics, and grape breeding along with innovations in technology as well as data analysis methods will definitely enable us to overcome these impediments and leverage the full potential of omics-based GS in grape polyploids.

## Author contributions

RB and MS drafted the manuscript. RB designed and created the figure. EF-C, LS and RS critically reviewed the manuscript and acquired funding. RB and MS revised the manuscript. All authors contributed to the article and approved the submitted version.
